# Assessing a peptidylic inhibitor-based therapeutic approach that simultaneously suppresses polyglutamine RNA- and protein-mediated toxicities in patient cells and *Drosophila*

**DOI:** 10.1242/dmm.022350

**Published:** 2016-03-01

**Authors:** Qian Zhang, Ho Tsoi, Shaohong Peng, Pan P. Li, Kwok-Fai Lau, Dobrila D. Rudnicki, Jacky Chi-Ki Ngo, Ho Yin Edwin Chan

**Affiliations:** 1 Laboratory of Drosophila Research, School of Life Sciences, Faculty of Science, The Chinese University of Hong Kong, Shatin, N.T., Hong Kong SAR, China; 2 Biochemistry Program, School of Life Sciences, Faculty of Science, The Chinese University of Hong Kong, Shatin, N.T., Hong Kong SAR, China; 3 Department of Psychiatry and Behavioral Sciences, Division of Neurobiology, Program of Cellular and Molecular Medicine, Johns Hopkins University School of Medicine, Baltimore, MD 21287, USA; 4 Cell and Molecular Biology Program, School of Life Sciences, Faculty of Science, The Chinese University of Hong Kong, Shatin, N.T., Hong Kong SAR, China; 5 Molecular Biotechnology Program, School of Life Sciences, Faculty of Science, The Chinese University of Hong Kong, Shatin, N.T., Hong Kong SAR, China

**Keywords:** Expanded-CAG RNA, Expanded-polyQ protein, Nucleolin, P3, Polyglutamine disease, QBP1, Spinocerebellar ataxia

## Abstract

Polyglutamine (polyQ) diseases represent a group of progressive neurodegenerative disorders that are caused by abnormal expansion of CAG triplet nucleotides in disease genes. Recent evidence indicates that not only mutant polyQ proteins, but also their corresponding mutant RNAs, contribute to the pathogenesis of polyQ diseases. Here, we describe the identification of a 13-amino-acid peptide, P3, which binds directly and preferentially to long-CAG RNA within the pathogenic range. When administered to cell and *Drosophila* disease models, as well as to patient-derived fibroblasts, P3 inhibited expanded-CAG-RNA-induced nucleolar stress and suppressed neurotoxicity. We further examined the combined therapeutic effect of P3 and polyQ-binding peptide 1 (QBP1), a well-characterized polyQ protein toxicity inhibitor, on neurodegeneration. When P3 and QBP1 were co-administered to disease models, both RNA and protein toxicities were effectively mitigated, resulting in a notable improvement of neurotoxicity suppression compared with the P3 and QBP1 single-treatment controls. Our findings indicate that targeting toxic RNAs and/or simultaneous targeting of toxic RNAs and their corresponding proteins could open up a new therapeutic strategy for treating polyQ degeneration.

## Abstract (precis)

***Drosophila* Collection:** We identified a novel peptidylic inhibitor against expanded CAG RNA toxicity. Our study highlights the importance of simultaneously targeting both toxic RNA and protein species in treating polyQ degeneration.

## INTRODUCTION

Polyglutamine (polyQ) diseases represent a group of dominantly inherited progressive neurodegenerative diseases (Orr and Zoghbi, 2007). These diseases are caused by genomic CAG trinucleotide repeat expansion in the coding region of the disease genes in which the CAG triplet repeats function as the codon for the amino acid glutamine. After gene transcription and protein translation, two primary toxic species – mRNA containing expanded CAG repeats and protein carrying an expanded polyQ domain – are produced in the neurons. These two mutant biomolecules induce neurotoxicity through multiple pathogenic pathways that lead to neurodegeneration (Fiszer and Krzyzosiak, 2013; Nalavade et al., 2013; Williams and Paulson, 2008). Recently, an additional RNA-dependent mechanism was reported by which toxic RNAs are translated into additional protein species with expanded homopolymeric amino acid tracts through the mechanism of repeat-associated non-ATG (RAN) translation initiation (Cleary and Ranum, 2014).

Ribosome biogenesis is essential for cellular protein synthesis. The ribosome is a ribonucleoprotein complex composed of ribosomal RNAs (rRNAs) and ribosomal proteins. Failure in rRNA transcription induces nucleolar stress, and cells undergo apoptosis. Thus, nucleolar stress is a cellular response designed to eliminate cells that fail to carry out efficient protein synthesis due to ribosome biogenesis defects (Boulon et al., 2010). A reduction in rRNA transcription leads to an imbalance of cellular levels of rRNAs and ribosomal proteins, and this results in an increased level of unassembled free ribosomal proteins, which are the proteinaceous components of the ribosome (Zhang and Lu, 2009). These free ribosomal proteins are targeted by the MDM2 E3 ubiquitin ligase for poly-ubiquitination and subsequent proteasome degradation. The engagement of MDM2 with free-ribosomal-protein degradation causes a cellular buildup of the p53 protein, which is a physiological substrate of MDM2. The cellular accumulation of p53 triggers the activation of mitochondrial-mediated apoptosis (Wang et al., 2014). Nucleolar stress response has been implicated in the pathogenesis of various neurodegenerative diseases (Parlato and Kreiner, 2013), including Alzheimer's and Parkinson's diseases, and amyotrophic lateral sclerosis. Our laboratory was the first to provide evidence that nucleolar stress is involved in the pathogenesis of polyQ diseases, including Machado Joseph disease (MJD; also known as SCA3) and Huntington's disease (HD) (Chan, 2014; Kreiner et al., 2013; Lee et al., 2011; Tsoi and Chan, 2013, 2014; Tsoi et al., 2012). We showed that expanded-CAG RNA interacts directly with the nucleolar protein nucleolin (NCL), and that this RNA-protein interaction prevents NCL from binding to the upstream control element (UCE) of the rRNA promoter. This then leads to UCE hypermethylation and downregulation of pre-45s rRNA transcription, which eventually triggers nucleolar-stress-induced apoptosis (Tsoi et al., 2012). We further showed that the overexpression of exogenous NCL protein inhibits UCE hypermethylation, restores pre-45s rRNA transcription and suppresses the nucleolar stress induced by expanded-CAG-RNA expression (Tsoi et al., 2012). These findings suggest that inhibition of the NCL–expanded-CAG-RNA interaction might offer a viable therapeutic strategy to suppress RNA toxicity in polyQ diseases. The human NCL protein carries four RNA recognition motifs (RRMs) (Ginisty et al., 1999), and our previous investigation pinpointed RRMs 2 and 3 as the interacting regions in NCL that mediate its binding to expanded-CAG RNA (Tsoi et al., 2012).

Peptidylic inhibitors have been demonstrated to disrupt the RNA-protein interaction, resulting in suppression of viral replication (Hamy et al., 1997). This prompted us to develop peptidylic inhibitors that could mitigate expanded-CAG-RNA toxicity. We scanned through a series of synthetic peptide sequences derived from RRMs 2 and 3 of the NCL protein, and identified a 13-amino-acid peptide, P3, which could bind directly and preferentially to expanded-CAG RNA (calculated *K*_D_=52.70±2.21 µM). Next, we further demonstrated that P3 disrupted the interaction between endogenous NCL protein and the expanded-CAG RNA. The introduction of the P3 peptide to cells expressing expanded-CAG RNA resulted in the restoration of the interaction between NCL and UCE, and the level of pre-45s rRNA expression. We further showed that P3 suppressed cell death in both cell and *Drosophila* disease models, and in MJD patient-derived fibroblasts. Our findings indicate that expanded-CAG-RNA toxicity can be targeted by peptidylic inhibitors. Finally, various peptidylic inhibitors (Arribat et al., 2013; Kazantsev et al., 2002; Mishra et al., 2012), including QBP1 (Nagai et al., 2000), have been reported to be capable of targeting polyQ-protein toxicity by inhibiting misfolding and aggregation of expanded-polyQ disease protein (Popiel et al., 2013). When P3 was co-administered with the polyQ-protein-toxicity inhibitor QBP1 (Nagai et al., 2000), the combined treatment of RNA- and protein-triggered toxicities led to even greater suppression of neurodegeneration *in vivo* in a *Drosophila* model of MJD. Our findings indicate that targeting toxic RNAs alone might be sufficient to elicit a significant therapeutic benefit, whereas the simultaneous targeting of both toxic RNAs and their corresponding toxic proteins is desirable to treat polyQ disease more efficaciously.

## RESULTS

### Identification of a peptide that prevents the binding of NCL to expanded-CAG RNAs

**Fig. 1.**
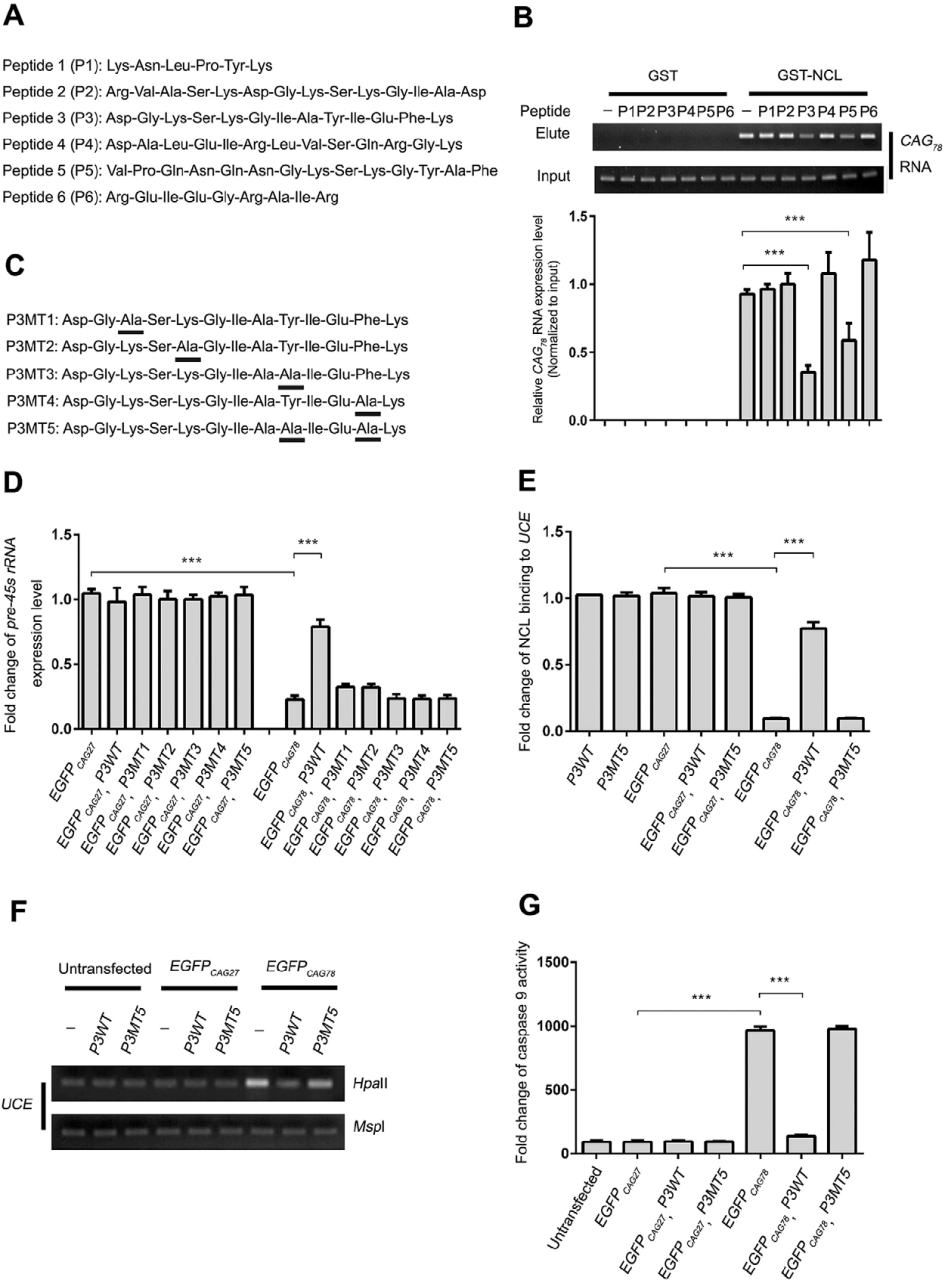
**Expression of *P3* suppressed nucleolar stress in cells expressed with expanded-CAG RNA.** (A) Amino acid sequence of nucleolin (NCL) peptides used in this study. (B) The P3 and P5 peptides disrupted the interaction between expanded-CAG RNA and NCL. After *in vitro* binding of CAG_78_ RNA and GST-NCL protein in the presence of NCL peptides, reverse-transcription PCR was performed to detect the binding of CAG_78_ RNA to GST-NCL. (C) Amino acid sequences of mutant (MT) P3 peptides. The mutated residues are underlined. (D) Expression of *P3WT* resumed the expression level of pre-45s rRNA in *EGFP_CAG78_* RNA-expressing HEK293 cells. Real-time PCR was performed to determine the expression level of pre-45s rRNA in cells co-transfected with *EGFP_CAG_* and *P3* constructs. (E) Expression of *P3WT* resumed the physical interaction between NCL and upstream control element (UCE) in *EGFP_CAG78_* RNA-expressing HEK293 cells. Chromatin immunoprecipitation was performed. Real-time PCR was performed to determine the amount of UCE in the immunoprecipitant. (F) Expression of *P3WT* resumed the DNA methylation status of UCE. ‘–’ represents cells that were transfected with *pcDNA3.1* empty vector. Genomic DNA was treated with either *Hpa*II or *Msp*I. *Hpa*II is a methylation-sensitive restriction enzyme, whereas *Msp*I is a methylation-insensitive restriction enzyme. The enzyme-treated DNA was used in PCR. Amplicon UCE was amplified. *Msp*I-treated samples were used as loading control. (G) Expression of *P3WT* suppressed caspase 9 activity in HEK293 cells expressing *EGFP_CAG78_* RNA. Experiments were repeated at least three times and data are expressed as mean±s.d. ***P<0.001.

We previously reported that expanded-CAG RNA triggered nucleolar stress in polyQ diseases (Tsoi and Chan, 2013; Tsoi et al., 2012). We showed that the overexpression of full-length Nucleolin (NCL) protein restores rRNA transcription and suppresses the pro-apoptotic events triggered by expanded-CAG RNA (Tsoi et al., 2012). This suggests that targeting the interaction between mutant RNA and NCL with inhibitors, such as peptides, represents a novel therapeutic direction. According to our previous observations (Tsoi and Chan, 2013; Tsoi et al., 2012), the information available on the structure of the RRMs of NCL protein [RCSB Protein Data Bank (PDB) ID: 2KRR] (Arumugam et al., 2010) and the RRM-RNA binding interface (Daubner et al., 2013), we synthesized six peptides (P1-P6) that covered the NCL RRM2 and RRM3 regions (Fig. 1A). The ability of individual peptides to interrupt the RNA-protein interaction between the *in-vitro*-transcribed expanded-CAG RNA, *MJD_CAG78_* (*MJD* is also known as *ATXN3*), and the purified GST-NCL protein was determined by a glutathione *S*-transferase pull-down assay (Tsoi et al., 2012). Two peptides, P3 and P5, were found to be capable of interfering with NCL binding to *MJD_CAG78_* RNA (Fig. 1B). We focused our subsequent investigation on P3 because this peptide was derived from NCL RRM2, whose structure was elucidated previously (Arumugam et al., 2010).

### P3 preferentially modulated rRNA transcription in cells that expressed expanded-CAG RNA

We next investigated whether P3 could mitigate expanded-CAG RNA toxicity in cells. Following overexpression of CAG RNA – *EGFP_CAG78_* – in HEK293 cells, a reduction in the level of pre-45S rRNA was observed compared to the cells overexpressing *EGFP_CAG27_* (Fig. 1D). However, when P3 was co-expressed, the levels of pre-45s rRNA were restored to 70% of the *EGFP_CAG27_* control. The effect was not due to P3 affecting the levels of *EGFP_CAG78_* RNA (Fig. S1). Furthermore, *P3* expression had no effect on the level of pre-45s rRNA in cells expressing the control construct *EGFP_CAG27_* (Tsoi et al., 2012) (Fig. 1D). Our data thus highlight the specificity of P3 action towards expanded-CAG RNA. Because rRNA transcription is mediated by RNA polymerase I, we further examined whether *P3* expression would affect the expression levels of genes that are transcribed by RNA polymerases II (*GAPDH*) and III (*U6* and *tRNA^met^*), and observed no change in RNA-polymerase-II- or III-mediated transcription in either *EGFP_CAG27_* or *EGFP_CAG78_* RNA-expressing cells (Fig. S2). This indicates that *P3* expression does not affect cellular gene transcription in general.

### Structure-activity relationship of P3

We next investigated the structure-activity relationship of P3. Because basic and aromatic side chains usually play crucial roles in protein/peptide–nucleic-acid interaction, we speculated that four residues in P3, namely Lys3, Lys5, Tyr9 and Phe12, are involved in RNA binding. These residues have previously been reported to play pivotal roles in RNA-protein (peptide) interactions (Iwakiri et al., 2012), including RRM-RNA interaction (Jenkins et al., 2011; Morozova et al., 2006). Hence, we generated five P3 point-mutant constructs (*P3MT1-5*; Fig. 1C). The *P3MT1-4* constructs each carry a single alanine substitution mutation at positions Lys3, Lys5, Tyr9 and Phe12, respectively, whereas *P3MT5* carries a Tyr9Ala Phe12Ala double mutation. Expression of the P3MT constructs was first confirmed by reverse transcription (RT)-PCR analysis, and their expression did not alter the levels of *EGFP_CAG78_* RNA (Fig. S1). Next, we examined the expression level of pre-45s rRNA in HEK293 cells co-transfected with both the *EGFP_CAG_* and individual P3MT constructs. In contrast to the P3WT positive control, rRNA transcription could not be restored via the expression of any of the P3MT constructs in *EGFP_CAG78_* RNA-expressing cells (Fig. 1D). This indicates that Lys3, Lys5, Tyr9 and Phe12 are all essential for P3 functioning. *P3MT5* was used as a negative control in our subsequent experiments.

### Mechanism of action of P3-mediated suppression of expanded-CAG-RNA toxicity

Expanded-CAG-RNA-induced nucleolar stress is initiated by a reduction in the binding of NCL to the UCE of the rRNA promoter, which results in UCE hypermethylation (Tsoi and Chan, 2014). We next hypothesized that the expression of *P3* prevents sequestration of NCL by expanded-CAG RNA, allowing it to resume its normal cellular role in rRNA transcription regulation. To test this, HEK293 cells were co-transfected with *EGFP_CAG78_* and *P3* constructs, and chromatin immunoprecipitation was performed to determine whether P3 restores the binding of endogenous NCL to UCE. Indeed, co-expression of *P3* restored the interaction between endogenous NCL and UCE in cells expressing *EGFP_CAG78_* RNA (Fig. 1E). No such effect was observed in the negative control, *P3MT5*. We further found that the expression of *P3WT*, but not *P3MT5*, suppressed *UCE* hypermethylation in *EGFP_CAG78_* RNA-expressing cells (Fig. 1F). Our findings indicate that P3 can effectively suppress expanded-CAG-RNA-induced nucleolar stress (Tsoi et al., 2012).

We previously demonstrated that expanded-CAG-RNA-induced apoptosis is mediated through the caspase pathway (Tsoi et al., 2012). We therefore examined the activity of distinct caspase pathways in cells that expressed *EGFP_CAG78_* RNA. The result showed that the activity of caspase 9, but not that of caspase 8, was elevated in HEK293 cells expressing expanded-CAG RNA (Fig. S3). When cells were co-transfected with *P3* and *EGFP_CAG78_*, caspase 9 activity was significantly suppressed when compared with the *EGFP_CAG78_*-transfected cells (Fig. 1G). This supports the idea that the intrinsic apoptotic pathway is involved in expanded-CAG-RNA toxicity, and is in line with our previous observations that expanded-CAG RNA induces mitochondrial cytochrome *c* release (Tsoi et al., 2012).

### P3 interacts directly with expanded-CAG RNA

**Fig. 2.**
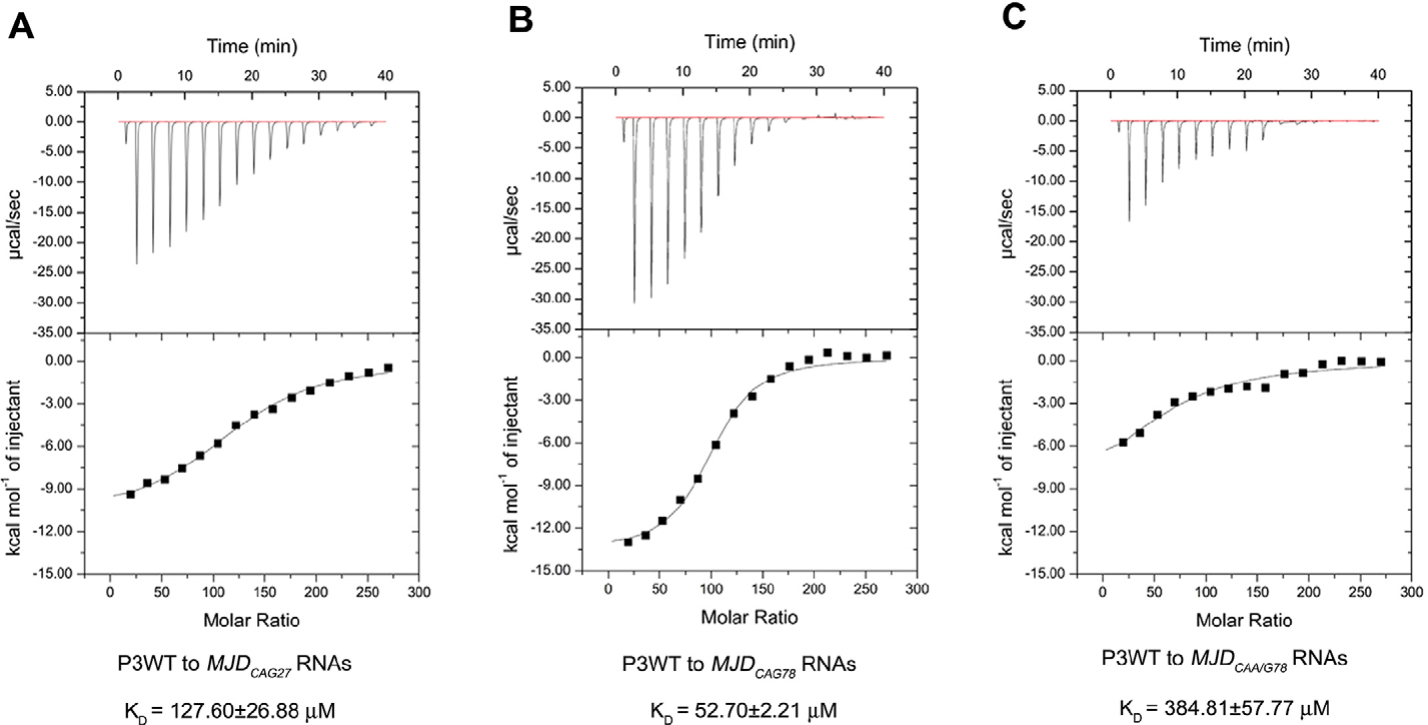
**P3 directly interacted with CAG-repeat-containing RNA.** Isothermal titration calorimetry study of the binding of synthetic P3WT peptide (13 mM) to CAG RNA (10 μM) *in vitro* transcribed from (A) *pcDNA3.1-MJD_CAG27_*, (B) *pcDNA3.1-MJD_CAG78_* and (C) *pcDNA3.1-MJD_CAA/G78_*. The top panel shows the raw thermogram and the bottom panel shows the binding isotherm fitted to a single-site model. The reported errors correspond to the s.d. of the fit. P3WT represents Peptide 3 wild type. Each experiment was repeated at least three times with consistent results obtained, and only representative graphs are shown.

We previously showed that NCL utilizes its RRM domains to interact with expanded-CAG RNA (Tsoi et al., 2012). We next tested whether P3, which is derived from NCL's RRM2, interacts physically with expanded-CAG RNA, by using isothermal titration calorimetry (iTC) (Li et al., 2009; Wong et al., 2014). We first showed that the synthetic wild-type P3 peptide associated with unexpanded *MJD_CAG27_* RNA with a *K*_D_ value of 127.60±26.88 µM (Fig. 2A). When compared with its interaction with *MJD_CAG27_* RNA, P3 bound to expanded *MJD_CAG78_* RNA with a lower *K*_D_ value (52.70±2.21 µM; Fig. 2B). This result indicates that P3 has a stronger interaction with RNA containing expanded-CAG-repeat RNA. In addition, we showed that P3 binding depends on the integrity of the CAG repeat: the peptide interacted weakly with CAA-interrupted CAG repeats in the context of the *MJD* transcript *MJD_CAA/G78_* (*K*_D_: 384.81±57.77 µM; Fig. 2C). Taken together, these results show that the P3 peptide interacts preferentially with long continuous CAG triple-repeat sequences. Our findings thus far demonstrate that P3 suppresses expanded-CAG-RNA toxicity (Fig. 1G) by binding directly to expanded-CAG RNA (Fig. 2), leading to subsequent release of NCL (Fig. 1B) and restoration of pre-45s rRNA transcription (Fig. 1D-F).

### Administration of synthetic P3 peptide suppressed expanded-CAG-RNA-induced cell death *in vitro*

**Fig. 3.**
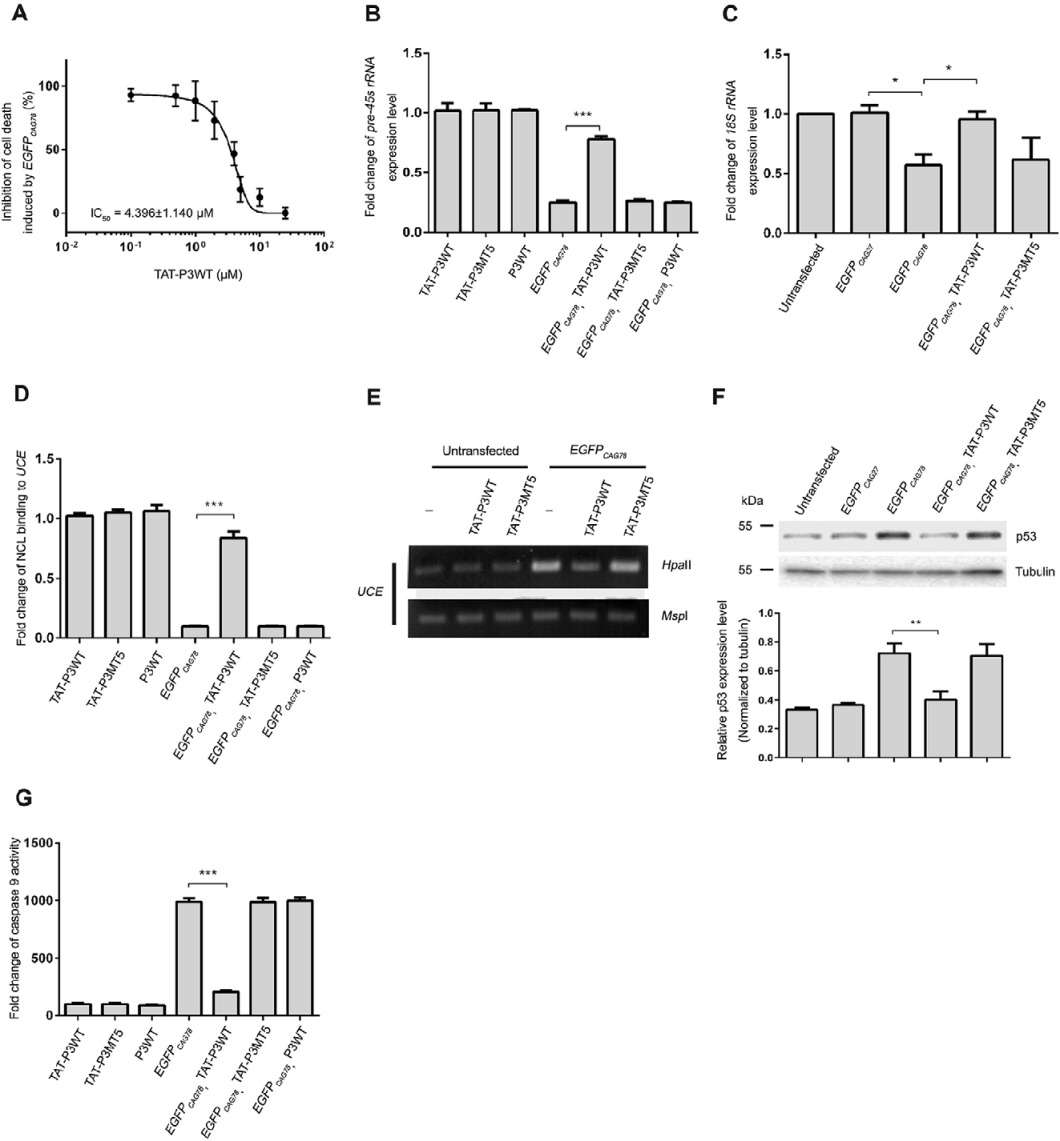
**P3 peptide treatment suppressed nucleolar stress in cells expressing expanded CAG RNA.** (A) Dose-dependent effect of synthetic TAT-P3WT on the inhibition of cell death in *EGFP_CAG78_* RNA-expressing HEK293 cells. A lactate dehydrogenase (LDH) cytotoxicity assay was performed. The IC_50_ value represents the concentration of TAT-P3WT that reduced LDH enzyme activity by 50% when compared with the no-peptide treatment control group. Data are expressed as mean±s.e.m. for at least three independent experiments. (B,C) Synthetic TAT-P3WT peptide (12 μM) treatment restored pre-45s rRNA (B) and 18S rRNA (C) levels in *EGFP_CAG78_* RNA-expressing HEK293 cells. Cells were treated with 12 μM of corresponding P3 peptides. Real-time PCR was performed to determine the level of pre-45s rRNA. ‘P3WT’ represents synthetic P3 peptide without the TAT fusion. This serves as a control to demonstrate that TAT-mediated intracellular delivery of P3 is crucial for its action. Experiments were repeated at least three times and data are expressed as mean±s.d. (D) Synthetic TAT-P3WT treatment resumed the interaction between NCL and UCE in *EGFP_CAG78_* RNA-expressing HEK293 cells. Following chromatin immunoprecipitation, real-time PCR was performed to determine the amount of UCE in the immunoprecipitant. Experiments were repeated at least three times and data are expressed as mean±s.d. (E) TAT-P3WT peptide treatment resumed the DNA methylation status of UCE in *EGFP_CAG78_* RNA-expressing HEK293 cells. ‘–’ indicates cells that were not treated with peptides. Genomic DNA was treated with either *Hpa*II or *Msp*I. *Hpa*II is a methylation-sensitive restriction enzyme, whereas *Msp*I is a methylation-insensitive restriction enzyme. Digested DNA was used in PCR. Amplicon UCE was amplified. *Msp*I-treated samples were used as loading control. Only representative gel photos are shown. (F) Synthetic TAT-P3WT peptide treatment inhibited p53 protein expression in *EGFP_CAG78_* RNA-expressing HEK293 cells. Western blotting was performed to determine the p53 expression level. Tubulin was used as a loading control. The experiment was repeated three times with consistent results obtained. Only representative blots are shown. (G) Synthetic TAT-P3WT peptide treatment suppressed cell death in HEK293 cells expressing *EGFP_CAG78_* RNA. Caspase 9 activity was determined. P3WT represents Peptide 3 wild type and P3MT5 represents P3 mutant 5. Experiments were repeated at least three times and data are expressed as mean±s.d. *P<0.05, **P<0.01 and ***P<0.001.

Cell-penetrating peptides (CPPs) have been widely used as a vehicle to enhance delivery of therapeutics across the cell membrane (Koren and Torchilin, 2012), including the peptidylic inhibitors of polyQ-protein toxicity QBP1 (Popiel et al., 2007, 2009) and htt^NT^ (Mishra et al., 2012). The TAT peptide is a CPP derived from the HIV-1 virus transactivator of transcription protein, which has been reported to mediate the translocation of proteins across the cell membrane (Frankel and Pabo, 1988; Green and Loewenstein, 1988). We therefore synthesized TAT-P3 fusion peptides (TAT-P3WT and TAT-P3MT5) (Fawell et al., 1994; Vives et al., 1997) and tested the effect of the fusion peptides on expanded-CAG-RNA toxicity. We first examined whether TAT-P3 treatment was capable of neutralizing expanded-CAG-RNA-mediated cytotoxicity in HEK293 cells and observed a dose-dependent reduction of cytotoxicity (Fig. 3A), as detected by the lactate dehydrogenase (LDH) cytotoxicity assay (Bañez-Coronel et al., 2012). The calculated maximal inhibitory concentration (IC_50_) value was 4.369±1.140 µM.

Next, we tested whether the effect of the TAT-P3 peptide on cytotoxicity is due to the suppression of NCL-mediated nucleolar stress (Tsoi et al., 2012). We first performed real-time PCR analysis to confirm that TAT-P3 treatment did not affect the expression level of *EGFP_CAG78_* RNA (Fig. S1). When compared with *EGFP_CAG78_* RNA-expressing HEK293 cells, *pEGFP_CAG78_*-transfected cells that were co-treated with the synthetic TAT-P3 peptide (12 µM) showed an increased level of pre-45s rRNA (Fig. 3B), 18S rRNA (Fig. 3C) and UCE-NCL interaction (Fig. 3D), as well as a reduction in UCE hypermethylation (Fig. 3E), p53 protein level (Fig. 3F) and caspase 9 activity (Fig. 3G). The above effects were not detected in cells co-treated with the TAT-P3MT5 negative-control peptide. Taken together, our results indicate that intracellular delivery of synthetic P3 peptide suppresses expanded-CAG-RNA-induced nucleolar stress and, subsequently, cell death. Although P3 physically interacts with cellular RNAs that carry short non-toxic CAG repeats (*MJD_CAG27_* RNA; Fig. 2A), it did not induce caspase activation in cells expressing the normal length of CAG repeats (Fig. 1G). We performed a set of control experiments to show that P3 does not act on the polyQ protein and that its effect is specific for expanded-CAG-repeat-containing RNAs, but not RNAs containing other trinucleotide-repeat expansions. First, we performed western blot analysis to examine whether TAT-P3 treatment would alter protein translation of *ataxin 2* (*ATXN2*) CAG mRNAs of different repeat lengths (22, 42, 55 and 72 CAGs). We observed no effect of TAT-P3 on the levels of ATXN2 proteins (Fig. S4A). Next, we found that the adult eclosion rate of wild-type *Drosophila* (Fig. S4B) and the viability of primary rat cortical neurons (Fig. S5) were not compromised when these models were treated with up to 1 mM and 25 µM of the TAT-P3 peptide, respectively. Finally, we showed that TAT-P3 had no effect on staurosporine (STS)-induced cell death *in vitro* (Fig. S6A), nor *in vivo* on toxicity induced by the expression of expanded-CUG (Garcia-Lopez et al., 2008) and -CGG (Jin et al., 2003) RNAs in *Drosophila* (Fig. S6B). Taken together, our findings demonstrate that the P3 peptide displays specificity for expanded-CAG-RNA-induced toxicity.

### Simultaneous suppression of RNA- and protein-induced toxicities in polyglutamine neurodegeneration

**Fig. 4.**
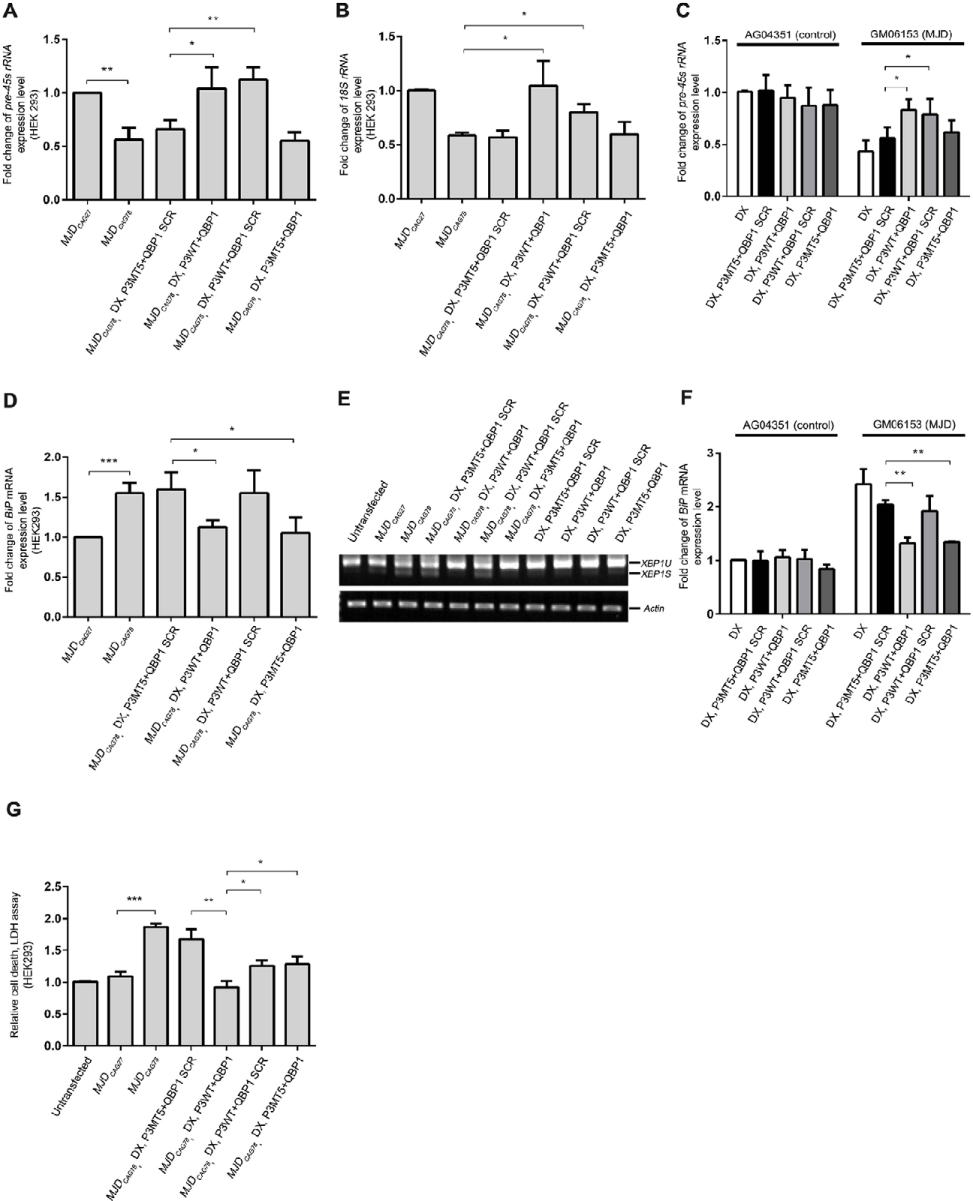
**Cellular transfection of synthetic P3 and QBP1 peptides suppressed expanded-CAG RNA-induced RNA toxicity and expanded-polyQ-protein-induced protein toxicity *in vitro*.** (A-F) Expression analyses of RNA and protein toxicity biomarkers in HEK293 cells (A,B,D,E) and MJD-patient-derived fibroblasts (GM06153) (C,F). Intracellular delivery of P3WT peptide (4 μM) through peptide transfection restored expression level of rRNAs in *MJD_CAG78_*-transfected HEK293 cells (A,B) and MJD-patient-derived fibroblasts (C). Delivery of QBP1 peptide (4 μM) reduced the induction level of *BiP* (D) and *XBP1S* (E) mRNAs in *MJD_CAG78_*-transfected HEK293 cells, and reduced the *BiP* level MJD-patient-derived fibroblasts (F). (G) Co-delivery of P3WT and QBP1 peptides (2 μM each) effectively inhibited cell death in *MJD_CAG78_*-transfected HEK293 cells. Lactate dehydrogenase (LDH) activity was assessed to measure cell death induced by expanded *MJD_CAG78_* RNA and MJDQ78 protein. An LDH assay was performed to measure the cytotoxicity. P3WT represents Peptide 3 wild type, P3MT5 represents P3 mutant 5, and QBP1 SCR represents scrambled control for QBP1. DX denotes DeliverX peptide transfection reagent; AG04351 denotes control human fibroblasts. For reverse-transcription PCR, only representative gels are shown and *actin* was used as loading control. Experiments were repeated at least three times and data are expressed as mean±s.d. *P<0.05, **P<0.01, ***P<0.001.

Because both expanded-CAG RNA and -polyQ protein contribute to neurotoxicity in polyQ degeneration (Fiszer and Krzyzosiak, 2013; Nalavade et al., 2013), we next tested a combined therapeutic approach to concomitantly target RNA and protein toxicities. In addition to CPP (Fig. 3), we also tested whether the peptide transfection reagent DeliverX (DX) (Deshayes et al., 2004) could be used to deliver synthetic peptides to cells and mitigate expanded-CAG-RNA toxicity. Our results showed that DX-mediated delivery of 4 µM of synthetic P3 peptide could effectively restore both the pre-45s rRNA level and UCE-NCL interaction, and suppress caspase 9 activity in our *EGFP_CAG78_-*RNA-toxicity-only cell model (Tsoi et al., 2012) (Fig. S7A-C). We further showed that DX-assisted intracellular delivery of P3 did not alter RNA-polymerase-II- and III-mediated gene expression (Fig. S8), indicating that the suppressive effect of the P3 peptide is specific for pre-45s rRNA transcription mediated by RNA polymerase I (Fig. S7A). After validating the DX-assisted peptide-delivery protocol, we utilized the *MJD_CAG78_* cell model (Tsoi et al., 2011) in our subsequent analyses because this model exhibits both expanded-CAG-RNA and -polyQ-protein toxicities. When *MJD_CAG78_*-transfected cells were treated with the synthetic P3 peptide, the levels of rRNA were restored to the *MJD_CAG27_* control level (Fig. 4A,B). Taken together, our results indicate that DX-mediated intracellular peptide targeting is effective in neutralizing expanded-CAG-RNA toxicity (Fig. 4A,B).

The QBP1 peptide (Nagai et al., 2000) is one of the most studied polyQ-protein-toxicity peptidylic inhibitors, and has been demonstrated to target disease-protein misfolding and aggregation (Popiel et al., 2013). Hence, we used the QBP1 peptide as a model polyQ-protein-toxicity inhibitor in our subsequent investigations. We first made use of the rRNA transcript as a readout to test whether the co-delivery of the P3 and QBP1 peptides would interfere with the suppression effect of P3 on RNA toxicity. Our results clearly showed that, when P3 was co-delivered with QBP1 or its scrambled control peptide (QBP1 SCR) to *MJD_CAG78_*-transfected cells, the rRNA level was restored back to the control level (Fig. 4A,B). In contrast, both P3MT5 control-peptide treatment groups (P3MT5+QBP1 and P3MT5+QBP1 SCR) failed to rescue the rRNA defects (Fig. 4A,B). This indicates that QBP1 co-delivery has no effect on the efficacy of P3. A similar result was obtained from MJD-patient-derived fibroblasts (Fig. 4C), further substantiating the application of peptide-based therapeutic interventions for expanded-CAG-RNA toxicity.

The binding immunoglobulin protein (BiP), also known as GRP-78 (Munro and Pelham, 1986), is a molecular chaperone responsible for protein refolding. Upregulation of BiP has been reported in polyQ diseases (Duennwald and Lindquist, 2008; Kouroku et al., 2002; Leitman et al., 2013). X-box binding protein 1 (XBP1) is a transcriptional factor that regulates chaperone gene expression, and its activation requires the excision of a 26-nucleotide fragment from the unspliced *XBP1* transcript (*XBP1U*) to generate the active spliced *XBP1S* mRNA for subsequent production of the functional XBP1 protein (Yoshida et al., 2001). To monitor polyQ-protein toxicity, we used both *BiP* gene induction and *XBP1* splicing as readouts and found that the expression of MJD_CAG78_ protein induced *BiP* transcription (Fig. 4D) and *XBP1S* production (Fig. 4E) in our *MJD_CAG78_* cell model. The DX-assisted delivery of QBP1, but not the QBP1 SCR scrambled control, reduced the cellular *BiP* expression and production of *XBP1S* (Fig. 4D,E; Fig. S9). This indicates that the QBP1 peptide suppresses polyQ-protein toxicity and that the suppression is not affected by the co-delivery of the P3 peptide. An effective suppression effect of QBP1 was also detected in MJD patient-derived fibroblasts (Fig. 4F).

When evaluating the overall inhibitory effects of the different treatment groups (Fig. 4A-F), the P3WT+QBP1 SCR group conferred only suppression on RNA toxicity, as shown by the restoration of rRNA transcript levels, whereas the P3MT5+QBP1 group solely mitigated protein toxicity, as determined by a reduction in *BiP* induction and *XBP1S* production (Fig. 4A-F). This indicates the respective suppression specificity of P3 and QBP1 in RNA and protein toxicities. In comparison to the P3WT+QBP1 SCR and P3MT5+QBP1 single-treatment groups, the P3WT+QBP1 co-treatment group was found to yield the most marked suppression of both expanded-CAG-RNA and -polyQ-protein toxicities as evidenced by both the significant restoration of rRNA defects and the reduction of *BiP* mRNA induction/*XBP1S* production in HEK293 cells (Fig. 4A,B,D,E), as well as in MJD-patient-derived fibroblasts (Fig. 4C,F). As a step further, we evaluated the efficacy of P3+QBP1 co-treatment in suppressing cytotoxicity in our *MJD_CAG78_* RNA/protein cell model. Based on the result of the LDH cytotoxicity assay, cells treated individually with either the functional P3WT (P3WT+QBP1 SCR) or QBP1 (P3MT5+QBP1) peptide yielded only a partial inhibition of cell death (Fig. 4G; Fig. S10). Intriguingly, the P3WT+QBP1 co-treatment group suppressed *MJD_CAG78_* RNA/protein-induced cell death more effectively when compared with the single-treatment groups (Fig. 4G). This demonstrates that P3WT+QBP1 co-treatment exerts an additive protective effect on *MJD_CAG78_* cell death conferred by both RNA and protein toxicities.

### P3/QBP1 peptide co-treatment effectively suppressed polyglutamine neurodegeneration *in vivo*

**Fig. 5.**
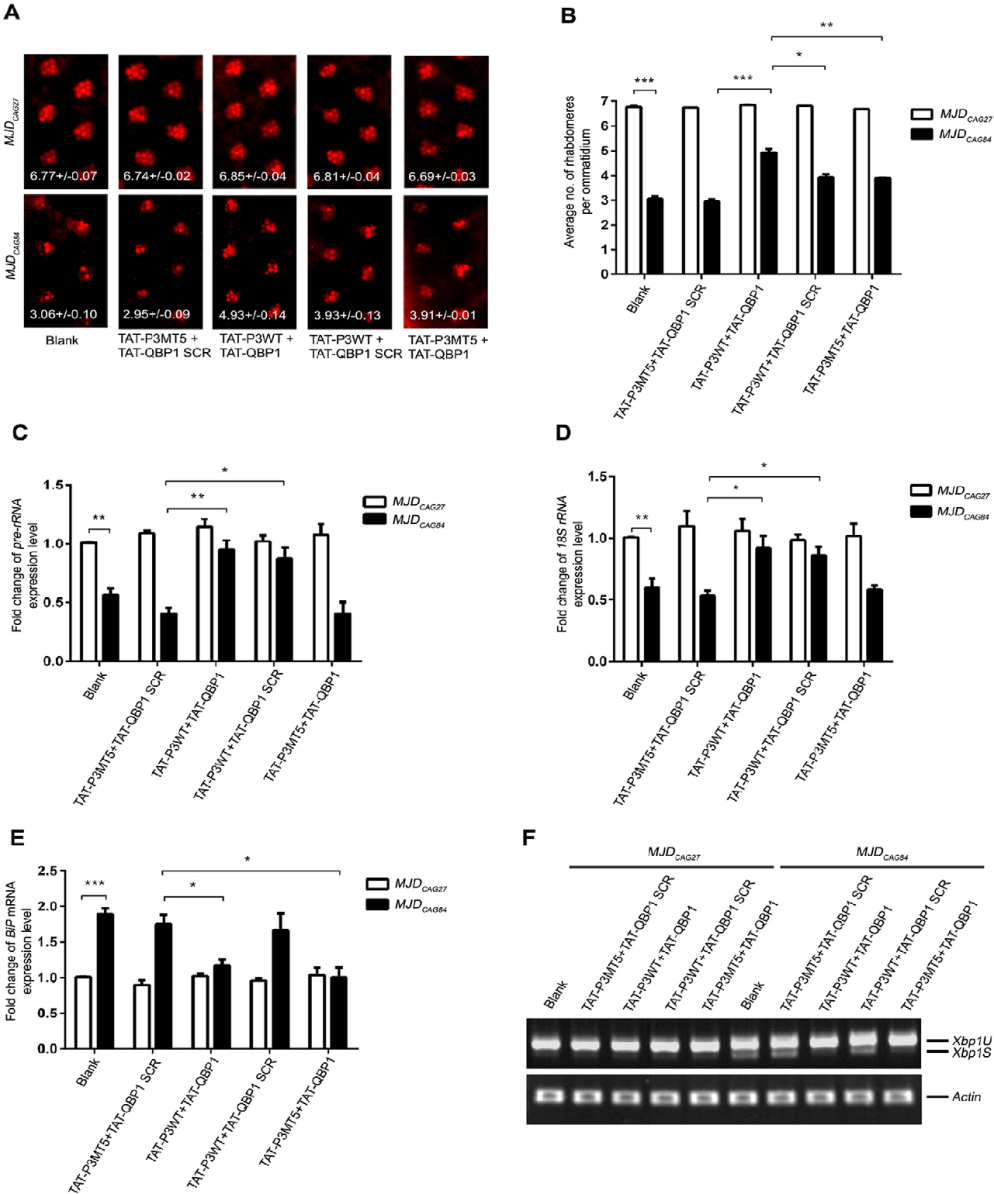
**P3/QBP1 co-treatment suppressed expanded-CAG-RNA-induced RNA toxicity and expanded-polyQ-protein-induced protein toxicity *in vivo*.** (A) Co-delivery of P3/QBP1 effectively suppressed flMJDQ84 neurodegeneration in *Drosophila*. When compared with the control groups, including blank, TAT-P3MT5/TAT-QBP1 SCR, TAT-P3WT/TAT-QBP1 SCR and TAT-P3MT5/TAT-QBP1, the transgenic *Drosophila flMJD_CAG84_* disease model co-treated with TAT-P3WT and TAT-QBP1 peptides (200 μM each) more significantly suppressed neurodegeneration *in vivo*. Pseudopupil assay was performed on 6-day-old adult flies. Numbers in the panels are the average number of rhabdomeres per ommatidium ±s.d. (B) Statistical analysis of panel A. Experiments were repeated at least three times and data are expressed as mean±s.d. (C-E) Real-time PCR analyses of pre-rRNA, 18S rRNA and *BiP* mRNA levels *in vivo*. Treatment of *flMJDQ84* flies with TAT-P3WT in combination with other peptides (200 μM each) resumed pre-rRNA (C) and 18S rRNA (D) levels. Similarly, the TAT-QBP1 treatment in combination with other peptides (200 μM each) reduced *BiP* mRNA expression level (E). Data are presented as fold change of the relative pre-rRNA or *BiP* expression levels compared with the untreated samples. Experiments were repeated at least three times and data are expressed as mean±s.d. *P<0.05, **P<0.01, ***P<0.001. (F) Reverse-transcription PCR analysis of *Xbp1* expression *in vivo*. Treatment of *flMJDQ84* flies with TAT-QBP1 in combination with other peptides (200 μM each) reduced *Xbp1S* level. Experiments were repeated at least three times, and only representative gels are shown. *actin* was used as loading control. The flies were of genotypes *w*; *gmr-GAL4 UAS-myc-flMJD_CAG27_/+*; +/+ and *w*; *gmr-GAL4/+; UAS-myc-flMJD_CAG84_/+*.

*Drosophila* has been used as an *in vivo* model to investigate peptidylic inhibitors of polyQ protein toxicity (Arribat et al., 2013; Kazantsev et al., 2002; Nagai et al., 2003). Peptide feeding was previously reported to be an effective way to deliver CPP-fusion QBP1 peptide to flies to mitigate polyQ protein toxicity, and it was demonstrated that 200 µM of QBP1 was capable of suppressing polyQ toxicity *in vivo* (Popiel et al., 2007). We utilized the full-length *MJD_CAG_* fly model, *flMJD_CAG27/84_* (Warrick et al., 2005), to investigate the combined suppression effect of P3 and QBP1. The expression of expanded *flMJD_CAG84_* RNA and flMJDQ84 protein caused severe retinal degeneration, which can be quantified by the pseudopupil assay (Chan et al., 2011) (3.06±0.10 rhabdomeres per ommatidium; Fig. 5A,B). We observed a mild but significant suppression of neurotoxicity in *flMJD_CAG84_* flies after they were treated with either the functional P3 (the TAT-P3WT+TAT-QBP1 SCR group; 3.93±0.13) or QBP1 (the TAT-P3MT5+TAT-QBP1 group; 3.91±0.01) peptidylic inhibitor (Fig. 5A,B). We also expressed the *flMJD_CAG84_* transgene using the pan-neural *Elav-GAL4* driver to test whether P3 could modulate expanded-CAG RNA toxicity in nervous tissues other than the eye. Pan-neural expression of the *flMJD_CAG84_* transgene caused adult lethality, and TAT-P3 treatment partially but significantly delayed *flMJD_CAG84_*-induced lethality in flies (Fig. S11). This indicates that the *in vivo* suppression effect of P3 is not simply confined to the photoreceptor neurons in the eye, but can further be extended to other nervous tissues.

We next determined whether a concurrent inhibition of both RNA and protein toxicities would yield an additive effect on the rescue of neurodegeneration *in vivo*. As expected, when *flMJD_CAG84_* flies were simultaneously treated with TAT-P3 and TAT-QBP1 peptides (200 µM each), a marked preservation of retinal integrity was observed as evidenced by a significant increase in the pseudopupil score (4.93±0.14) when compared with the single-treatment groups (Fig. 5A,B). More importantly, no deleterious effect was observed when TAT-P3 and TAT-QBP1 were co-administered *in vivo*, as indicated by the retinal integrity of the *flMJD_CAG27_* control flies (Fig. 5A,B). This finding is consistent with our cell-based toxicity (Fig. S10) and animal lethality (Fig. S4) investigations, in which P3 and QBP1 peptidylic inhibitors did not elicit any dominant toxicity effect under our experimental conditions. We further found that treating flies with both non-functional peptidylic inhibitors, TAT-P3MT5 and TAT-QBP1 SCR, did not cause any suppression of neurodegeneration (2.95±0.09; Fig. 5A,B). This demonstrates that the TAT CPP component of the peptides did not contribute to the phenotypic suppression.

To further confirm that P3+QBP1 co-treatment mitigates both expanded-CAG-RNA and -polyQ-protein toxicities, we examined the expression levels of rRNA (Larson et al., 2012) (Fig. 5C,D), *BiP* (Chow et al., 2015) (Fig. 5E) and *Xbp1S* (Ryoo et al., 2007) (Fig. 5F) in *flMJD_CAG_* flies treated with TAT-P3 and/or TAT-QBP1. Real-time PCR analysis demonstrated a marked restoration of rRNA transcript levels (Fig. 5C,D) in animals treated with TAT-P3 in combination with either TAT-QBP1 or TAT-QBP1 SCR (Fig. 5C,D). This confirms the suppression effect of the P3 peptide on expanded-CAG-RNA-mediated nucleolar stress induction *in vivo*. We next investigated the rescue effect of QBP1 on polyQ-protein toxicity (Popiel et al., 2007) in our fly model. We first demonstrated that the expression level of the protein-misfolding biomarkers *BiP* (Fig. 5E) and *Xbp1S* (Fig. 5F) were induced in the *flMJD_CAG84_* flies when compared to that of the *flMJD_CAG27_* control. This confirms that *BiP* and *Xbp1S* are reliable markers for monitoring protein toxicity in polyQ degeneration *in vivo*. When *flMJD_CAG84_* flies were treated with TAT-QBP1 peptide either in combination with TAT-P3WT or TAT-P3MT5, we observed suppression of *BiP* induction (Fig. 5E) and *Xbp1* splicing (Fig. 5F). We further showed that TAT-P3 and TAT-QBP1 treatment did not affect the protein expression of the unexpanded polyQ MJD disease protein (Fig. S12). Upon TAT-QBP1 administration, we also observed that the stacking gel-residing SDS-insoluble expanded polyQ MJD protein was partially diminished (Fig. S12). Furthermore, our findings illustrate that the mitigating effects of P3WT (Fig. 5C,D) and QBP1 (Fig. 5E,F; Fig. S12) on RNA and protein toxicity, respectively, were not influenced by the other co-administered peptide. Taken together, our data suggest that the simultaneous targeting of RNA and protein cellular toxicities using peptide agents is a viable approach for developing effective treatments for polyQ diseases.

## DISCUSSION

There is growing evidence that both mutant polyQ proteins (Williams and Paulson, 2008) and transcripts that encode the proteins (Fiszer and Krzyzosiak, 2013; Nalavade et al., 2013) contribute to the pathogenesis of polyQ diseases. Over the past decade, several peptidylic inhibitors have been developed to target polyQ protein toxicity, many of which have demonstrated promising therapeutic potential (Arribat et al., 2013; Kazantsev et al., 2002; Mishra et al., 2012; Nagai et al., 2000). However, the development of inhibitors for expanded-CAG-RNA-mediated neurotoxicity has lagged behind. Peptidylic and small-molecule inhibitors represent the two major groups of therapeutics for combating polyQ neurotoxicity, and both demonstrate significant therapeutic potential (Bauer and Nukina, 2009; Shao and Diamond, 2007). Of the two, peptidylic inhibitors are generally considered more selective (Cirillo et al., 2011). This property is particularly important in polyQ disease because a successful treatment requires an agent that can discriminate between mutant RNA/protein species and their wild-type counterparts.

**Fig. 6.**
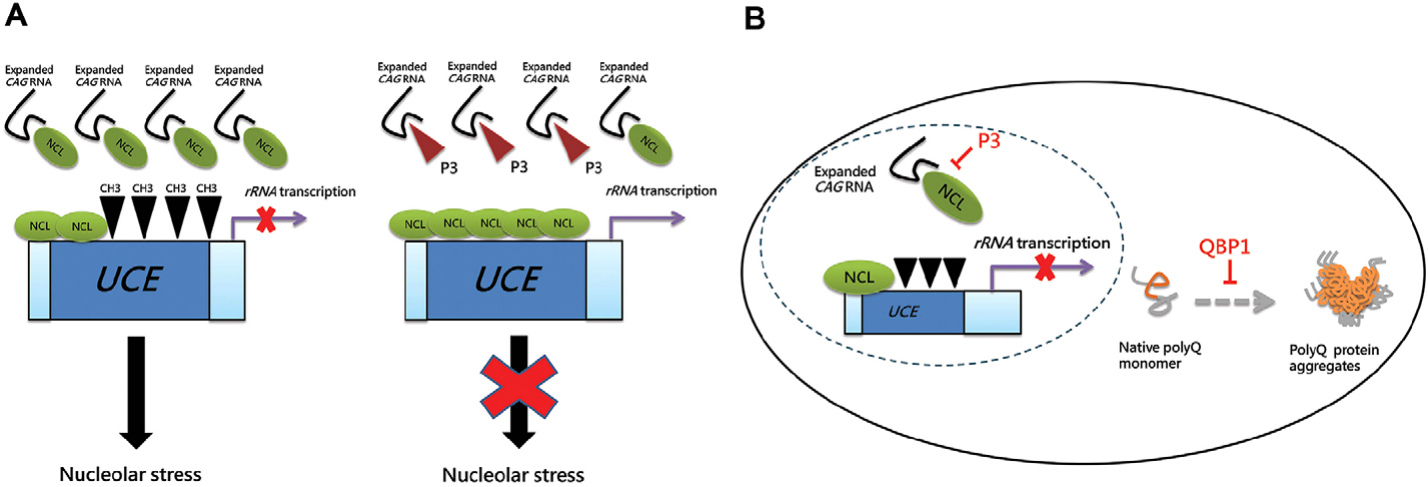
**Schematic diagram illustrating mechanism of actions of P3 and QBP1 in suppressing RNA and protein toxicities of polyQ degeneration.** (A) P3 suppressed expanded-CAG-RNA-induced nucleolar stress. (B) Suppression of RNA toxicity and protein toxicity utilizing the P3-QBP1 combination treatment strategy. CH3, methyl group.

Our previous investigation of RNA toxicity in polyQ diseases (Tsoi et al., 2012) led us to identify P3, a 13-amino-acid peptide derived from NCL (Fig. 1), which is capable of neutralizing the nucleolar stress induced by expanded-CAG RNA *in vitro* (Figs 1, 3 and 4) and *in vivo* (Fig. 5). The P3 peptide preferentially binds to CAG RNA within the pathogenic repeat range, and diminishes the interaction between NCL and the mutant RNA (Fig. 6A). This leads to the reduction of UCE hypermethylation, restoration of pre-45s rRNA transcription, and blockade of nucleolar stress induction (Fig. 6A). We demonstrated that P3 is effective in suppressing RNA toxicity in both an artificial expanded-CAG RNA (Figs 1 and 3) and specific polyQ disease (Figs 4 and 5) models. This suggests that P3 is a generic peptidylic inhibitor against CAG-RNA toxicity.

To date, multiple parallel pathogenic mechanisms have been reported to contribute to expanded-CAG-RNA toxicity (Evers et al., 2014; Martí and Estivill, 2013; Nalavade et al., 2013; Tsoi and Chan, 2014). In our study, we determined the empirical IC_50_ value of our expanded CAG-RNA-toxicity peptidylic inhibitor P3 based on cell death inhibition in *EGFP_CAG78_* RNA-expressing cells (∼4 µM; Fig. 3A). Recently, a small-molecule compound, D6, was identified that is capable of correcting the pre-mRNA splicing in an HD-patient-derived cell model (Kumar et al., 2012). Both D6 and P3 are capable of inhibiting particular RNA-toxicity-associated molecular pathogenic mechanisms, namely RNA mis-splicing for D6 (Kumar et al., 2012) and nucleolar stress for P3 (Figs 1 and 3). More importantly, both studies unequivocally demonstrate that expanded-CAG RNA toxicity can be targeted therapeutically. It would be of interest to further determine whether P3 and D6 suppress RNA toxicity through targeting the same set of cellular pathogenic events, or whether each has its own distinct set of suppression mechanisms. Although our study describes the identification of the first peptidylic inhibitor that targets expanded-CAG-RNA toxicity, the prototypic P3 sequence could be further subjected to peptide-engineering modifications (Ramos-Martín et al., 2014), such as N- and C-terminal truncation (Tomita et al., 2009), to improve its potency.

The QBP1 peptide is a well-characterized peptidylic inhibitor of polyQ protein toxicity (Nagai et al., 2000). β-sheet conformation transition of polyQ protein has been shown to be responsible for triggering protein toxicity in polyQ degeneration (Nagai et al., 2007), and QBP1 was reported to suppress protein toxicity by attenuating polyQ β-sheet conformation transition (Hervás et al., 2012; Nagai et al., 2007). In addition, QBP1 was found to be capable of inhibiting polyQ protein aggregation (Nagai et al., 2000). Both β-sheet conformation transition and aggregation of polyQ protein intimately associate with protein misfolding, and molecular chaperones are a class of cellular proteins responsible for promoting proper protein folding. Several previous observations have demonstrated that the expression level of multiple heat shock protein (HSP) genes are upregulated in polyQ diseases (Huen and Chan, 2005; Huen et al., 2007; Tagawa et al., 2007), and such gene induction events are considered to be a cellular protective mechanism aiming to neutralize protein toxicity through promoting refolding of the polyQ disease protein. As one of the members of the molecular chaperone family, BiP protein levels were previously reported to be upregulated in polyQ disease (Duennwald and Lindquist, 2008; Kouroku et al., 2002; Leitman et al., 2013). In this study, we further showed that *BiP* gene expression as well as the spliced form of *XBP1* mRNA, *XBP1S*, were induced in both *in vitro* and *in vivo* (Figs 4 and 5) conditions. This and previous findings emphasize a global activation of molecular chaperone machinery, including HSPs such as *BiP*, to combat toxicity that associates with polyQ protein misfolding. Although QBP1 single-treatment already resulted in a notable attenuation of cell death (Fig. 4E) and neurodegeneration (Fig. 5B), a P3/QBP1 co-treatment clearly led to a more complete suppression.

One challenging issue in therapeutic intervention of polyQ diseases is the delivery of inhibitors to the cellular targets: neurons in the central nervous system (CNS). An increasing number of peptide therapeutics have entered clinical-trial phases in recent years (Kaspar and Reichert, 2013); one of the reasons could be the development of newly emerging peptide-drug technologies such as cell-penetrating peptides (Fosgerau and Hoffmann, 2015). GRN1005 is a peptide-drug conjugate for treating advanced brain tumors, and it was found that intravenous administration of GRN1005 to patients resulted in the shrinkage of brain metastases (Kurzrock et al., 2012). This suggests intravenous delivery as a possible route for the delivery of P3 and QBP1 to the CNS in polyQ patients. In addition, a short peptide sequence derived from the rabies virus glycoprotein was reported to be able to deliver proteins to the CNS (Fu et al., 2012). In our study, we showed that the attachment of the TAT cell-penetrating peptide (Frankel and Pabo, 1988; Green and Loewenstein, 1988) to both P3 and QBP1 did not alter their therapeutic properties (Figs 3 and 5). This opens up the possibility of further modifying the cell-penetrating peptide moiety of P3 and QBP1 for achieving CNS-targeting. However, the distinctive pathophysiology of the degenerating neurons in polyQ patients might make the outcome of the peptide delivery strategies less predictable. Nevertheless, our results indicate that an effective treatment strategy for polyQ disease might require simultaneous targeting of toxic RNA and protein species.

## MATERIALS AND METHODS

### Construction of plasmids

The *pcDNA3.1-MJD_CAG27_*, *pcDNA3.1-MJD_CAG78_*, *pcDNA3.1-MJD_CAA/G78_*, *pEGFP_CAG27_* and *pEGFP_CAG78_* constructs were reported previously (Li et al., 2008; Tsoi et al., 2012). To generate the *pcDNA3.1-myc-ATXN2_CAG22/42/55/72_* constructs, *ATXN2* DNA fragments containing 21 bp upstream and 105 bp downstream of the CAG repeats were PCR amplified from patient brain samples and cloned into *pcDNA3.1(-)myc-His A* vector using *Eco*RV enzyme. To generate peptide expression constructs, oligonucleotide linkers were employed. All DNA oligos were ordered from Life Technologies. The *P3WT* linker was generated by annealing the following oligos: *P3WTF* 5′-AATTCATGGATGGTAAGTCAAAGGGTATCGCTTACATCGAGTTCAAGTAAC-3′ and *P3WTR* 5′-CGAGTTACTTGAACTCGATGTAAGCGATACCCTTTGACTTACCATCCATG-3′. The *P3MT1* linker was generated by annealing the following oligos: *P3MT1F* 5′-AATTCATGGATGGTGCTTCAAAGGGTATCGCTTACATCGAGTTCAAGTAAC-3′ and *P3MT1R* 5′-TCGAGTTACTTGAACTCGATGTAAGCGATACCCTTTGAAGCACCATCCATG-3′. The *P3MT2* linker was generated by annealing the following oligos: *P3MT2F* 5′-AATTCATGGATGGTAAGTCAGCTGGTATCGCTTACATCGAGTTCAAGTAAC-3′ and *P3MT2R* 5′-TCGAGTTACTTGAACTCGATGTAAGCGATACCAGCTGACTTACCATCCATG-3′. The *P3MT3* linker was generated by annealing the following oligos: *P3MT3F* 5′-AATTCATGGATGGTAAGTCAAAGGGTATCGCTGCTATCGAGTTCAAGTAAC-3′ and *P3MT3R* 5′-TCGAGTTACTTGAACTCGATAGCAGCGATACCCTTTGACTTACCATCCATG-3′. The *P3MT4* linker was generated by annealing the following oligos: *P3MT4F* 5′-AATTCATGGATGGTAAGTCAAAGGGTATCGCTTACATCGAGGCTAAGTAAC-3′ and *P3MT4R* 5′-TCGAGTTACTTAGCCTCGATGTAAGCGATACCCTTTGACTTACCATCCATG-3′. The *P3MT5* linker was generated by annealing the following oligos: *P3MT5F* 5′-AATTCATGGATGGTAAGTCAAAGGGTATCGCTGCTATCGAGGCTAAGTAAC-3′ and *P3MT5R* 5′-TCGAGTTACTTAGCCTCGATAGCAGCGATACCCTTTGACTTACCATCCATG-3′. The annealed linkers were ligated to *pcDNA3.1* vector digested with *Eco*RI and *Xho*I.

### Synthesis of peptides and CAG RNAs

All peptides were purchased from GenScript USA Inc. The P3 peptide sequences are shown in Fig. 1A,C and the QBP1 sequence is shown as follows: SNWKWWPGIFD. Amino acid sequence of the TAT cell-penetrating peptide used in our study was YGRKKRRQRRR (Popiel et al., 2007). Sequences of the TAT-fusion peptides used in this study are as follows: TAT-QBP1, SNWKWWPGIFD-YGRKKRRQRRR; TAT-QBP1 SCR, WPIWSKGNDWF-YGRKKRRQRRR; TAT-P3WT, YGRKKRRQRRR-DGKSKGIAYIEFK and TAT-P3MT5, YGRKKRRQRRR-DGKSKGIAAIEAK. The purity of peptides used in cell experiments and *in vitro* binding was over 95%. Desalted peptides were used in *Drosophila* feeding assays. All RNAs were synthesized using the MEGAscript^®^ kit (Ambion) as previously described (Tsoi et al., 2012), and the *MJD_CAG27_*, *MJD_CAG78_* and *MJD_CAA/G78_* RNAs were transcribed from linearized *pcDNA3.1-MJD_CAG_* constructs (Tsoi et al., 2011).

### Cell culture, plasmid transfection and peptide transfection

Normal human fibroblasts (AG04351) and MJD-patient-derived fibroblasts (GM06153) were obtained from the Coriell Institute for Medical Research (Camden, NJ, USA). Both HEK293 cells and fibroblasts were cultured at 37°C with 5% CO_2_ in DMEM supplemented with 10% FBS and 1% penicillin-streptomycin. Primary rat cortical neurons were isolated and cultured as previously described (Lau et al., 2008). Transient transfection of HEK293 cells was performed using Lipofectamine 2000 (Life Technologies). Peptides were delivered to HEK293 cells using the DeliverX (DX) Peptide Transfection kit (Affymetrix) 4 h after DNA transfection. Four micromolar of peptides were used to transfect cells, except for the LDH cytotoxicity assay, in which 2 µM of peptides were used. For the TAT-fusion peptide treatment, 12 µM of TAT-P3WT, TAT-P3MT5, TAT-QBP1 and TAT-QBP1 SCR peptides were added directly to the culture medium at the time of DNA transfection unless otherwise stated. At least two batches of independently synthesized peptides were used in the experiments.

### *In vitro* binding assay

Purified nucleolin protein (GST-NCL) was purchased from Abnova (Taiwan), and the control GST protein was expressed and purified as mentioned in Tsoi et al. (2012). One hundred micromolar of corresponding peptides were added to the *CAG_78_* RNA/GST-NCL mixture. The reaction mixture was incubated at 4°C with end-to-end rotation for 2 h. The beads were then washed three times with 1 ml of binding buffer. Each wash was conducted for 10 min at 4°C. After the washing steps, 100 µl of GST elution buffer (20 mM Tris-Cl, pH 7.4, 20 mM glutathione) was used to elute the protein-RNA complex. RNA extraction was performed. Reverse-transcription PCR was performed to amplify the CAG amplicon with primers *CAGF* 5′-AAAAACAGCAGCAAAAGC-3′ and *CAGR* 5′-TCTGTCCTGATAGGTCC-3′. Band intensity was measured using ImageJ (Schneider et al., 2012). Each experiment was repeated at least three times, with consistent results obtained.

### RNA extraction, reverse-transcription PCR and real-time PCR

RNA was extracted from cells or ten 6-day-old adult fly heads by Trizol reagent (Life Technologies), and 1 µg of purified RNA was then used for reverse-transcription using the ImPromII™ Reverse Transcription System (Promega). Random hexamer (Roche) was used as primers in reverse transcription. The amplicon of *actin* was amplified by primers *actinF* 5′-TGTGCAAGGCCGGTTTCGC-3′ and *actinR* 5′-CGACACGCAGCTCATTGTAG-3′; the amplicons of *P3WT* and *P3MT1-5* were amplified by primers *P3F* 5′-TAATACGACTCACTATAGGG-3′ and *P3R* 5′-TAGAAGGCACAGTCGAGG-3′; the amplicon of *CAG_78_* RNA was amplified by primers *CAGF* 5′-AAAAACAGCAGCAAAAGC-3′ and *CAGR* 5′-TCTGTCCTGATAGGTCC-3′; the amplicon of *XBP1S* for human was amplified by primers *XBP1SF* 5′-GGAGTTAAGACAGCGCTTGG-3′ and *XBP1SR* 5′-ACTGGGTCCAAGTTGTCCAG-3′; and the amplicon of *Xbp1S* for *Drosophila* was amplified by primers *Xbp1SF* 5′-CAACAGCAGCACAACACCAG-3′ and *Xbp1SR* 5′-AGACTTTCGGCCAGCTCTTC-3′. Taqman gene expression assays were performed on an ABI 7500 Real-time PCR system and data were analyzed as previously described (Tsoi et al., 2012). The following probes were used: *pre-45s rRNA* (Assay ID: AILJIZM), *pre-rRNA* (Assay ID: AIMSG5*U*), *18S rRNA* (Assay ID: Hs03928985_g1), human *GAPDH* (Assay ID: Hs99999905_m1), *Drosophila GAPDH* (Assay ID: Dm01841186), *U6* (Assay ID: AII1MM6), *tRNA^met^* (Assay ID: AIN1FB2), *UCE* (Assay ID: AIHSOGY) and *actin* (Assay ID: Hs99999903_m1), human *BiP/GRP78* (Assay ID: Hs99999174_m1), *Drosophila BiP/GRP78* (Assay ID: Dm01813415-g1), *EGFP* (Assay ID: Mr04097229_mr) and *ATXN3* (Assay ID: Hs01026440_g1). Each experiment was repeated at least three times.

### Western blotting

All protein samples were resolved on 12% SDS-PAGE, and detected using the following antibodies: 7F5 (Cell Signaling Technology; 1:1000) for p53, 9B11 (Cell Signaling Technology; 1:2000) for myc-tagged proteins. Tubulin was detected by E7 (Developmental Studies Hybridoma Bank; 1:5000). Each experiment was repeated at least three times, and comparable results were obtained.

### Chromatin immunoprecipitation and *Hpa*II methylation assays

Chromatin immunoprecipitation was performed according to Tsoi et al., 2011, 2012. Antibody used was anti-nucleolin 3G4B2. To perform the *Hpa*II methylation assay, genomic DNA was extracted from cells, followed by digestion with 2 units of *Hpa*II or *Msp*I (New England Biolabs) for 4 h at 37°C. The DNA products were incubated at 85°C for 15 min to heat-inactivate the restriction enzymes. The resulting DNA products were amplified by PCR. Amplicon of the human UCE was amplified by *UCEF*, 5′-CGTGTGTCCTTGGGTTGACC-3′ and *UCER*, 5′-CGCGTCACCGACCACGCC-3′. Each experiment was repeated at least three times, with consistent results obtained.

### Caspase activity assays

Caspase activity was measured using the Caspase-Glo^®^8 and Caspase-Glo^®^9 assay systems (Promega) following the manufacturer's instructions. TNF-related apoptosis-inducing ligand (TRAIL) served as a positive control for the caspase 8 activity assay, whereas staurosporine (STS) served as a positive control for the caspase activity 9 assay. EnVision^®^ Multilabel Reader (PerkinElmer) was used to measure the luminescence. Each sample was measured in duplicates, and each experiment was repeated at least three times.

### Isothermal titration calorimetry binding assay

Experiments were carried out using a MicroCal iTC200 isothermal titration calorimeter (GE Healthcare) at 25°C. Data were analyzed using the Origin^®^ scientific plotting software version 7 (Microcal Software Inc.). All RNAs and peptides were dissolved in binding buffer (20 mM MOPS, pH 7.0; 300 mM NaCl). The concentration of RNA was estimated with appropriate extinction coefficients at 260 nm on a Nanodrop 2000 (Thermo Scientific). A reference power of 8 μcal/s was used with an initial 0.5 µl of injection of peptide followed by 2.5 µl for all subsequent titration points, with a 60 s initial equilibrium delay and 150 s pause between injections. The samples were stirred at a speed of 1000 rpm throughout the experiment. The thermal titration data were fitted to the ‘one binding site model’ to determine the dissociation constant (*K*_D_). At least two batches of independently synthesized peptides were used in the experiments. Each experiment was repeated at least three times with consistent results obtained.

### Lactate dehydrogenase (LDH) cytotoxicity assay and IC_50_ determination

Human embryonic kidney 293 (HEK293) cells were seeded on a 24-well plate at a density of 0.5×10^5^, and *pcDNA3.1-MJD_CAG27/78_* or *pEGFP_CAG78_* DNA construct was used to transfect the cells. Four hours after DNA transfection, peptide transfection was performed as follows: P3WT-QBP1, P3WT-QBP1 SCR, P3MT5-QBP1 and P3MT5-QBP1 SCR (2 μM each). For STS (Feng and Kaplowitz, 2002) treatment, cells were treated with 1 µM of STS in conjunction with 12 µM TAT-P3WT. LDH enzyme activity in the cell culture medium was measured 24 h (for STS experiment) or 72 h (for *pEGFP_CAG78_* transfection experiments) post-treatments using the Cytotox 96 non-radioactive cytotoxicity assay (Promega). Each experiment was repeated at least three times, with consistent results obtained.

To detect the effect of P3WT on inhibiting cell death in *EGFP_CAG78_* RNA-expressing HEK293 cells, the LDH assay was employed. A density of 0.5×10^5^ HEK293 cells were transfected with *pEGFP_CAG78_* and various amounts of the TAT-P3WT peptide – 0.1, 0.5, 1, 2, 4, 5, 10 and 25 μM – were then added to individual culture wells. Seventy-two hours after treatment, LDH enzyme activity in the cell culture medium was measured as described before. Experimental groups were normalized to the untransfected control. After normalization, data were analyzed using the dose response-inhibition curve (nonlinear regression-variable slope) to determine the IC_50_ value (Prism6 software, GraphPad Software, Inc.).

### *Drosophila* genetics, peptide feeding and assays

Flies were raised at 21.5°C or 25°C on cornmeal medium supplemented with dry yeast. Fly lines bearing *UAS-flMJD_CAG27_* and *UAS-flMJD_CAG84_* (Warrick et al., 2005) were gifts from Professor Nancy Bonini (University of Pennsylvania, USA). The *UAS-EGFP-CGG_90_* (Jin et al., 2003) and *UAS-(CTG)_480_* (Garcia-Lopez et al., 2008) fly lines were obtained from Professors Stephen Warren (Emory University, USA) and Rubén Artero Allepuz (Universitat de València, Estudi General, Spain), respectively*.* The *gmr-GAL4*, *elav-GAL4* and *Oregon R* fly lines were obtained from the Bloomington *Drosophila* Stock Center. For the pseudopupil assay, third instar larvae were fed with 200 µM of respective peptides dissolved in 2% sucrose solution for 2 h and then continued to culture in standard fly food at 21.5°C (Chau et al., 2006). The pseudopupil assay was performed on 6-day-old adult flies as mentioned previously (Wong et al., 2008). Images were captured by a SPOT Insight CCD camera controlled by the SPOT Advanced software (Diagnostic Instruments Inc.). Image processing was performed using the Adobe Photoshop CS software (Adobe). Each experiment was repeated at least three times (*n*=10 fly heads), and consistent results were obtained. For lifespan analysis, third instar larvae were fed with 200 µM of TAT-P3WT or TAT-P3MT5 (dissolved in 2% sucrose solution) for 2 h and then continued to culture in standard fly food at 25°C. Two days after eclosion, 10-15 adult flies were allocated to individual fresh non-drug-containing food vials. At least 120 flies were analyzed per treatment group. The flies were transferred to fresh vials every 3 days during the whole course of the experiment, and the number of surviving flies was counted every 3 days. Survival rate was calculated as area under survival curve followed by one-way ANOVA analysis. For the wild-type adult eclosion test, *Oregon R* third instar larvae were fed with 500 µM or 1 mM of TAT-P3WT peptide dissolved in 2% sucrose solution for 2 h, and then continued to culture in standard fly food at 25°C. Adult eclosion rate was calculated as the number of adult flies divided by the number of larvae examined. Each experiment was repeated three times (*n*=60 larvae). Two batches of independently synthesized peptides were used in the experiments.

### Statistical analyses

Data were analyzed by one-way ANOVA followed by post-hoc Tukey test. *, ** and *** represent *P*<0.05, *P*<0.01 and *P*<0.001, respectively, which are considered statistically significant.
